# Combination of *Bacillus tequilensis* with difenoconazole to control pear black spot and the related synergistic mechanism

**DOI:** 10.3389/fmicb.2024.1405039

**Published:** 2024-06-04

**Authors:** Qiuyan Bi, Fen Lu, Jie Wu, Xiangyu Liu, Xiuying Han, Wenqiao Wang, Jianjiang Zhao

**Affiliations:** Key Laboratory of Integrated Pest Management on Crops in the Northern Region of North China, IPM Center of Hebei Province, Institute of Plant Protection, Hebei Academy of Agricultural and Forestry Sciences, Ministry of Agriculture, Baoding, China

**Keywords:** *Alternaria alternata*, synergistic mechanisms, combined efficacy, field applications, pear

## Abstract

**Background:**

Pear black spot (PBS) is caused by *Alternaria alternata* and causes severe damage worldwide. It is particularly important to screen for synergistic fungicide combinations to address issues associated with the low efficacy of biocontrol agents, high dosage requirements and poor sustained effectiveness of chemical fungicides.

**Methods:**

*In vitro* and *in vivo* studies were performed to determine the efficacy of a treatment for this important disease. Additionally, transcriptomic and metabolomic analyses were performed to determine the main molecular and biochemical mechanisms involved in the interaction.

**Results:**

*Bacillus tequilensis* 2_2a has a significant synergistic effect with difenoconazole, causing hyphal entanglement and spore lysis and inhibiting the formation of PBS lesions *in vitro*. In the field, the control effect of the combination was greater than 95%. The pathways associated with the synergistic effect on the mycelia of *A. alternata* were divided into two main types: one included glycolysis, oxidative phosphorylation, and MAPK signal transduction, while the other included glycolysis, the TCA cycle, coenzyme A biosynthesis, sterol synthesis, and fatty acid degradation. Both types of pathways jointly affect the cell cycle. The main functions of the key genes and metabolites that have been verified as being affected are glucose synthesis and oxidative respiration, as well as citric acid synthesis, acetyl-CoA synthesis, and sterol synthesis. Both functions involve intracellular pyridine nucleotide metabolism and adenine nucleotide transformation.

**Conclusion:**

This study helps to reveal the synergistic mechanisms underlying the combined efficacy of biological and chemical agents, providing a scientific basis for field applications.

## 1 Introduction

China is one of the three major centers of pear cultivation worldwide. Its pear cultivation area and yield are the highest in the world, accounting for approximately two-thirds of global pear production (Teng et al., 2021). Pear black spot (PBS) can reduce annual pear production by 20–50%, and in severe cases, the reduction can reach 80% (Bi et al., 2023). The pathogenic fungus *Alternaria alternata* can produce more than 70 toxic metabolites, posing a direct threat to the health (Aremu et al., 2004). At present, chemical control is still the main method used in pear production, but there are problems associated with this approach, such as those caused by the single use of fungicides, the use of large amounts of chemical fungicides, and the frequent application of chemical fungicides (Percival et al., 2009). The use of chemical pesticides leads to the accelerated development of pathogen resistance and excessive accumulation of pesticide residues. The synergistic control of PBS using chemical and microbial agents is highly important for reducing the use of chemical pesticides, reducing pollution, and improving ecological and environmental health.

*Bacillus* spp. are important resources for the development of microbial pesticides and have been widely applied (Tafifet et al., 2020; Wang et al., 2023). These bacteria exert biocontrol effects by inhibiting the growth, reproduction, and infectivity of pathogens (Etesami et al., 2023). The microbial fungicide “Weijunjing,” with the *B. subtilis* NCD-2 strain as the active ingredient, has good field control effects against cotton wilt (Guo et al., 2014). The microbial fungicide Serenada, with *B. subtilis* QST713 as the active ingredient, is used to control diseases such as powdery mildew, gray mold, and downy mildew (Dewen, 2013). The antibacterial effect is also one of the main mechanisms by which *Bacillus* spp. exert their biocontrol effects (Cawoy et al., 2015). The antibacterial substances produced by *Bacillus* spp. include mainly lipopeptides, proteins, and volatile antibacterial substances. Fengycin can effectively inhibit the growth of filamentous fungi, causing their tips or branches to swell and rupture, disrupting the structure of cell membranes (Wise et al., 2014) and causing leakage of cellular contents, localized hyphal damage (Ongena and Jacques, 2008), and cell death. Research has shown that *Bacillus* spp. can also be mixed with chemical pesticides (Krishnamoorthy et al., 2017; Ji et al., 2019), and these agents have been applied in the control of major pear diseases, such as in the synergistic control of PBS by *B. subtilis* and pyrimethanil (Chang et al., 2010).

Difenoconazole is a sterol synthesis inhibitor that exhibits strong and rapid action but poor sustained effects. Difenoconazole has a single site of action, and long-term and single use of this agent will inevitably increase the risk of pathogen resistance. The widespread use of difenoconazole in pear orchards to control pear diseases has shown the potential to reduce sensitivity. Previous studies have shown that this fungicide can inhibit the growth of fungal hyphae, spore germination, and expansion of disease spots. In production, the problem of poor sustained effectiveness is generally solved by combining difenoconazole with endothermic fungicides, for example, by combining difenoconazole and the strobilurin-based chemical fungicide azoxystrobin to control PBS (Wang et al., 2016).

One of the strategies to improve the efficacy of biocontrol agents and promote the sustained efficacy of chemical fungicides is to screen for combinations with strong synergistic control effects and sustained efficacy. Regardless of whether biocontrol agents are used alternately or in combination with chemical agents, they are inevitably present in environments where chemical agents exist. The *B. tequilensis* 2_2a strain is an excellent biocontrol strain that was screened by the Plant Protection Research Institute of the Hebei Academy of Agriculture and Forestry (Bi et al., 2021). This strain has the ability to antagonize PBS caused by *A. alternata*. This article describes a systematic study of (i) the combined effects of *B. tequilensis* 2_2a and the chemical fungicide difenoconazole on *A. alternata* and their control effects on PBS and (ii) the potential synergistic mechanism involved. The identification of key targets will provide a theoretical basis for the combined application of microbial and chemical fungicides in production and provide technical support for the development of efficient chemical fungicides for the control of PBS at reduced dosages in China.

## 2 Materials and methods

### 2.1 Antifungal biocontrol strain, fungicides, and pathogenic strain preparation

The antifungal biocontrol strain *B. tequilensis* 2_2a was granted a Chinese invention patent, announcement number CN114231444B. Mycelia of *B. tequilensis* 2_2a were picked and cultured in Luria–Bertani (LB) medium at 180 rpm and 30°C for 36 h. Under a microscope with an oil immersion lens at a magnification of 1,000×, the density of the fermentation broth of *B. tequilensis* was adjusted to 1 × 10^9^ cfu/mL. A spectrophotometer was used to measure the absorbance (OD_660_), which was adjusted to 0.8.

The fungicides used were 95% difenoconazole (Li′er Chemical Co., Ltd., Qingdao) and 10% difenoconazole (water-dispersible granules; Syngenta Crop Protection Co., Ltd., Switzerland); the difenoconazole was dissolved in acetone to prepare a 1.0 × 10^4^ μg • mL^−1^ stock solution.

The pathogenic strain *A. alternata* JS-1 was obtained from the laboratory of the Jiangsu Academy of Agricultural Sciences. *A. alternata* JS-1 was inoculated onto potato dextrose agar (PDA) plates and grown at 25°C for 5 days.

### 2.2 Synergistic antifungal activity of *Bacillus tequilensis* and difenoconazole and control effect of *Bacillus tequilensis* and difenoconazole

#### 2.2.1 Determination of the antifungal activity against the mycelia of *Alternaria alternata*

To measure the synergistic antifungal activity of microbial and chemical fungicides, the cup plate drilling method was used (Wang et al., 2022). PDA plates (containing 1 μg • mL^−1^ difenoconazole) were prepared, and four holes were drilled per plate. Then, 20 μL of a 1 × 10^5^ cfu/mL suspension of *B. tequilensis* was added to each hole. Simultaneously, a control group was established in which the PDA plates contained only difenoconazole at the same concentration; a control group was established without addition of the *B. tequilensis* suspension; and a blank control group was established that was inoculated with *A. alternata* hyphal disks. The experiment was conducted twice with four independent replicates. The plates were continuously incubated at 25°C, and the colony diameter of *A. alternata* was measured according to Bi et al. (2023) when the *A. alternata* in the blank control had grown on 2/3 of the culture dish, approaching full coverage of the culture dish. Moreover, at the edges of the target pathogenic fungi, mycelia of *A. alternata* were observed at a magnification of 200× using a microscope (Keyence, Osaka, Japan). The calculation formulas for mycelial inhibition were as described by Bi et al. (2023).

#### 2.2.2 Determination of the antifungal activity against the spores of *Alternaria alternata*

*Alternaria alternata* was cultured on PDA plates at 25°C for 14 days to produce spores, and the spore suspension was prepared according to Kong et al. (2022). Then, 50 μL of the *A. alternata* spore suspension (1 × 10^3^ cfu/mL) was evenly spread onto a 1.5% water agar (WA) plate (containing 1 μg • mL^−1^ difenoconazole), and 20 μL of the *B. tequilensis* spore suspension (1 × 10^5^ cfu/mL) was added. Moreover, on PDA plates containing the same concentration of difenoconazole, a control without the addition of the *B. tequilensis* suspension was established, as was a blank control with only the *A. alternata* spore suspension. All treatments were incubated at 25°C in the dark for 8 h. The experiment was conducted twice and repeated independently four times. By using an optical microscope with 400x magnification (Olympus Tokyo, Hataya, Japan), approximately 50 *A. alternata* spores were examined. The formulas used to calculate the spore germination rate and spore inhibition rate were as described by Yang et al. (2023).

#### 2.2.3 Determination of the antifungal activity against PBS on detached fruits

Pear fruit of the same size and maturity were selected and fully immersed in *B. tequilensis* (1 × 10^5^ cfu/mL), difenoconazole (1 μg • mL^−1^), or *B. tequilensis* + difenoconazole (1 × 10^5^ cfu/mL + 1 μg • mL^−1^). Pears were soaked in sterile water as a blank control. After the surface water had dried off, a wound with a diameter of 2 mm was established by pricking, and a 5 mm diameter disk of *A. alternata* was used for inoculation (Pan et al., 2019). For each treatment, 15 fruit were tested, and the test was repeated four times. The samples were sealed in a transparent plastic box, held at 25°C for 12 h, and incubated in the dark with 60 to 70% relative humidity for 7 days. The lesion area was measured. The *in vitro* antifungal activity (%) = [(blank control lesion area - treatment lesion area)/blank control lesion area] × 100.

#### 2.2.4 Field testing of the synergistic effect against PBS

In 2020, 2021, and 2022, field effect experiments were conducted in the “Huangguan” pear orchard in Liangshan village, Changli city, Hebei Province. The row spacing of the 10- to 12-year-old pear trees was 4 m × 4 m. In this orchard, PBS occurred every year. The first application of the fungicide was carried out before the occurrence of the disease during the young-fruit stage of pear trees. The application dates were June 4, 15, and 28, 2020; June 2, 15, and 26, 2021; and June 5, 15, and 27, 2022. The following recommended dosages were used: *B. tequilensis* (1 × 10^7^ cfu/mL), difenoconazole (20 μg • mL^−1^), and *B. tequilensis* + difenoconazole (1 × 10^7^ cfu/mL + 20 μg • mL^−1^). Water was used as a blank control. Each repetition included four pear trees. The distance between different treatments was 4 m. All the different treatments were repeated four times. A 3WBD-20 high-voltage electric spray (Taizhou Luqiao Industry Co., Ltd.) was used for spraying, and the sprayer pressure was 0.30 ~ 0.40 MPa. One litre of solution was sprayed on each tree. As the fruit approached maturity, a survey was conducted to determine the disease index. The disease survey methods and calculation formulas for the synergistic effect of *B. tequilensis* and difenoconazole were as described by Bi et al. (2023).

### 2.3 Transcriptomic and metabolomic analysis of the synergistic effect against *Alternaria alternata*

Shake cultivation of *A. alternata* mycelia from different treatment groups was performed for transcriptomic and metabolomic analyses. Three 5-mm-diameter *A. alternata* disks were treated with 54 mL of PD culture medium supplemented with the treatment agent and shaken at 25°C and 180 r/min for 36 h. Then, 6 mL of *B. tequilensis* (1 × 10^6^ cfu/mL), difenoconazole (10 μg • mL^−1^), or *B. tequilensis* + difenoconazole (1 × 10^6^ cfu/mL + 10 μg • mL^−1^) was added; sterile water was added to the blank control group. After coculture for 3 days, the mixtures were rinsed with sterile water and vacuum filtered to obtain *A. alternata* mycelia from the different treatments. All the different treatments were repeated four times. Using the Novozan Total RNA Extraction Kit (Eastern) ® Super, RNA was extracted from the above samples. Principal component analysis (PCA) was performed by using the vst function in DESeq2 to calculate the distance between samples. DESeq2 software (Love et al., 2014) was used to analyze the differential gene expression between samples, and genes whose expression differed between different samples by more than twofold were considered differentially expressed. The differentially expressed genes (DEGs) were subjected to Kyoto Encyclopedia of Genes and Genomes (KEGG) enrichment analyses. To control the proportion of false positives, the *p* value from the hypothesis test was corrected using padj. The experimental criteria were |log2(fold change)| > 1 and padj<=0.05.

The extraction and detection of metabolic components from *A. alternata* mycelia were performed according to Bi et al. (2023). PCA was performed by using the vst function in DESeq2 to calculate the distance between samples. The number of shared and unique metabolites in different groups was calculated using a Venn diagram. Similarly, DESeq2 software (Love et al., 2014) was used to analyze the differentially abundant metabolites between samples, and the statistical test parameters were consistent with those described for RNA. The KEGG_v94.2 database was used to perform KEGG functional pathway analysis (*p* < 0.05).

Based on the synergistic pathways identified via transcriptome and metabolic correlation analysis, a core regulatory network model of *A. alternata* treated with a combination of *B. tequilensis* and difenoconazole was constructed.

### 2.4 Verification of the main target genes and metabolic components involved in the synergistic effect

#### 2.4.1 Verification of the main target genes involved in the synergistic effect

The main target genes were selected for RT–PCR analysis to test the consistency of the RNA-seq gene expression patterns. Total RNA was separated and reverse transcribed according to Bi et al. (2023). The primers for qRT–PCR were designed using Primer Premier 6 (Table 1 lists some of the primers used; all the primers used are listed in Supplementary Table 1). A real-time fluorescence quantitative PCR system (Applied Biosystems, Foster, United States) was used for recording fluorescence changes, and the results were verified for four independent replicates. The 2-_ΔΔ_Ct formula for calculating the relative expression level was as described by Wu et al. (2020).

**Table 1:** List of qRT–PCR primers used for fluorescence quantification.

Gene name	Gene function description	Forward primer (5′-3′)	Reverse primer (5′-3′)
CC77DRAFT_1004828 (down)	Glycolysis/Gluconeogenesis	TGTTGGATCAGCTGTGGGAT	ACAACTCTGCCCTGTAGCTT
CC77DRAFT_1005031 (down)	Citrate cycle (TCA cycle)	TTCCCCAAAGATCTCCACCC	GCAGCAATGGTAGGCAGTTT
CC77DRAFT_1024999 (down)	Oxidative phosphorylation	TACTGAGCACTGGGGAAAGG	CTGTGTCTTTCTCCATCGCG
CC77DRAFT_948756 (down)	cytochrome P450	ATGGCTTCTAAGGATCCGGG	GACTGTGTTGGCTAGGAGGT
CC77DRAFT_928877 (down)	Fatty acid biosynthesis	GTGTCTTTGCTAGCTACGCC	ACCAGAAACCATACCGACGT
CC77DRAFT_990455 (down)	Steroid biosynthesis	TTCGACCCTCTATACGCCAC	GCGTCCATTGGCTCATGTAG
CC77DRAFT_947345 (down)	acetyl-CoA synthetase-like protein	CATTGTCTACGCTCCACTGC	CACCCTTTTGCCCTCTTTCC
CC77DRAFT_1030172 (down)	MAPK signaling pathway	ACCCAAGAGCCAGATCGAAA	GATCCTACAACGGCGACAAC
CC77DRAFT_1098683 (down)	Cell cycle	CTTGAAGATGCAGACGAGGC	GTTAGTGCTGTGCTTTGCCT
CC77DRAFT_985514 (down)	MFS general substrate transporter	CCATCAGCCGTATCCTGGAT	TACCGTTCCCCAGTCCATTC
CC77DRAFT_1039035 (down)	ABC transporter	TGCACTGACACCATCTCCAT	TGACAACCATGCCACCAATG
CC77DRAFT_1000211 (up)	NAD(P)-binding protein	AATGGGGATGTCTGCTTCGA	ACAACACCAATTTCGACCGG
CC77DRAFT_1024752 (down)	NADPH dehydrogenase	CTCGCAAATGGAGCCTCTTC	CCAGCCGTTCATCTCCTTTG
CC77DRAFT_486143 (up)	AMP deaminase	CACACACGTTCACCACTCAG	CAGAGTCAGCAGATTGTCGC
CC77DRAFT_942416 (down)	ATP-dependent RNA helicase	TCATTTTCGTCAGGACCCGA	TTGATGACCATGGTGACGGA
CC77DRAFT_938423 (down)	ADP-ribose pyrophosphatase	CTCCTCGGATGCAAAATGGG	ACGGGTGGTCTGAATTGGAT
CC77DRAFT_1063831 (down)	Valine, leucine, and isoleucine biosynthesis	CACTGTGAGTAGACGCATGC	ACCAGCATACCTTCTCCACC
CC77DRAFT_1061793(down)	Glycine, serine, and threonine metabolism	TGGAAGCTGCTGGATCTCAA	TTCACCCTTTGCGACCTTTG

#### 2.4.2 Verification of the main metabolic components involved in the synergistic effect

##### 2.4.2.1 Detection of glycolysis (extracellular acidification rate, ECAR) and mitochondrial respiration (oxygen consumption rate, OCR) in *Alternaria alternata*

The ECAR and OCR are indicators used to evaluate glycolysis, mitochondrial function, and oxidative phosphorylation in cells and are commonly used in research on cellular energy metabolism.

For ECAR measurement, *A. alternata* was inoculated and cultured on PDA plates for 14 days to produce spores. The spores were washed twice with phosphate-buffered saline, after which they were washed twice with detection buffer, and a spore suspension with a concentration of 5–6 × 10^5^ cfu/mL was obtained. Then, 100 μL of the spore suspension was added to each well of the cell culture plate for automated detection (Solarbio Technology Co., Ltd., Beijing, China). The wells were washed with 100 μL of P61 detection buffer. The detection buffer was removed after the second wash, and 90 μL of fresh detection buffer was added. Then, 10 μL of BBcellProbe was added to each well containing the P61 acidified fluorescent probe and mixed thoroughly. Equal volumes (10 μL) of filtered *B. tequilensis* (1 × 10^6^ cfu/mL), difenoconazole (10 μg • mL^−1^), and *B. tequilensis* + difenoconazole (1 × 10^6^ cfu/mL + 10 μg • mL^−1^) were added separately, and sterile water without *B. tequilensis* was used as the blank control. A Seahorse energy metabolism analyzer was subsequently used to measure the changes in pH and calculate the ECAR.

OCR measurement was performed as follows: a spore suspension with a concentration of 5–6 × 10^5^ cfu/mL was used. Four microlitres of the BBoxiProbe VIII R01 oxygen fluorescence probe was added to each well of the cell culture plate (product number 100777004, USA) and mixed thoroughly. Equal volumes (4 μL) of filtered *B. tequilensis* (1 × 10^6^ cfu/mL), difenoconazole (10 μg • mL^−1^), and *B. tequilensis* + difenoconazole (1 × 10^6^ cfu/mL + 10 μg • mL^−1^) were added separately, with sterile water without *B. tequilensis* used as the control. Then, 100 μL of oxygen blocking solution was added, and a Seahorse energy metabolism analyzer was used to measure the changes in the dissolved oxygen content.

##### 2.4.2.2 Changes in the citric acid content in *Alternaria alternata*

A total of 0.1 g of mycelia from each treatment group was ground with 1 mL of buffer solution to obtain a homogeneous slurry. The mixture was centrifuged at 10,000 × g for 10 min, after which the supernatant was transferred to a new centrifuge tube for testing. All processes were carried out on ice. The citric acid content and citrate synthase activity were measured and calculated according to Bi et al. (2023) with the MitoCheck^®^ Citric Acid Content Assay Kit/MitoCheck^®^ Citrate Synthase Activity Assay Kit (Cayman Co., Ltd., Beijing, China).

##### 2.4.2.3 Changes in the intracellular pyridine nucleotide content in *Alternaria alternata*

NADP^+^, NADPH, NAD^+^ and NADH were specifically extracted from 0.1 g of mycelia from the different treatment groups and detected using the NADP^+^/NADPH and NAD^+^/NADH Quantification Colorimetric Kit (BioVision Co., Ltd., Shanghai, China). Mycelia (0.1 g) from the different treatment groups were resuspended with 0.50 mL of acidic extraction solution, sonicated for 1 min with 2-s pulses at 1-s intervals, covered tightly, boiled in water for 5 min, cooled in an ice bath, and centrifuged at 10000 × g at 4°C for 10 min. The supernatant (200 μL) was transferred to a new centrifuge tube, and an equal volume of alkaline extract was added to neutralize it. The mixture was centrifuged at 10000 × g and 4°C for 10 min, after which the supernatant was collected. The NADPH/NADP^+^ concentration and NADH/NAD^+^ ratio were calculated according to Bi et al. (2023).

##### 2.4.2.4 Changes in the intracellular adenine nucleotide content in *Alternaria alternata*

A ZIKER reagent kit (Solarbio Technology Co., Ltd., Beijing, China) was used for the separation and quantitative detection of ATP, ADP, and AMP in mycelia from different treatment groups via high-performance liquid chromatography (HPLC) (Thermo Fisher Corporation, MA, United States). A total of 0.1 g of mycelia from each of the different treatment groups was placed in centrifuge tubes. The samples were frozen in liquid nitrogen for 60 s and stored at −20°C. Then, 10 mL of 0.60 mol L^−1^ HClO_4_ was added to a centrifuge tube with frozen mycelia and stirred for 10 min using a magnetic stirrer. After centrifugation at 10000 × g for 10 min, the supernatant was collected. An additional 10 mL of 0.60 mol L^−1^ HClO_4_ was added to another centrifuge tube containing frozen mycelia and mixed thoroughly for 10 min, and the supernatant was collected after centrifugation. The two supernatants were mixed in a 25 mL volumetric flask, and the volume was adjusted to 25 mL with 0.60 mol L^−1^ HClO_4_. Ten milliliters of the prepared solution was collected, and the pH was adjusted to 7.0 with 0.80 mol L^−1^ KOH. After incubation at 4°C for 30 min, the crystalline KClO_4_ was removed through a 0.22 μm filter membrane, and the solution was diluted to 25 mL with pH 7.0 phosphate buffer for analysis.

##### 2.4.2.5 Changes in acetyl-CoA content in *Alternaria alternata*

Acetyl-CoA extraction was performed according to Duan et al. (2020). The mixture was centrifuged at 12,000 rpm and 4°C for 10 min and placed on ice for testing. The UV spectrophotometer was preheated for 30 min, and the wavelength was set to 340 nm. The reagent was brought to room temperature (25°C). The following components were added sequentially to a 1 mL quartz colorimetric dish: sample, 80 μL; Reagent 2, 40 μL; Reagent 3, 40 μL; Reagent 4, 640 μL. The four components were mixed well; the absorbance A_1_ was immediately recorded at 340 nm, and the absorbance A_2_ was recorded after 10 min. The _△_A value was calculated as A_2_-A_1_. The acetyl-CoA content (nmol/g) was calculated according to Ma et al. (2023).

##### 2.4.2.6 Changes in the sterol content in *Alternaria alternata*

Mycelia (0.1 g) from each treatment group were collected. The ultrasonic cell pulverization method was used for sterol extraction (Huo et al., 2021). Chromatographically pure methanol was used as the solvent for ultrasonic extraction. HLPC analysis was conducted for sterol with the following conditions: 250 mm × 46 mm (id) stainless steel column; column temperature: room temperature; flow rate: 1.0 mL/min; injection volume: 10 μL; mobile phase: 100% methanol (chromatographic alcohol); UV detection wavelength, 282 nm.

### 2.5 Data statistics and analysis

The antifungal activity and control effects, as well as the results verifying the metabolite contents, were calculated, and the average values were subjected to analysis of variance (ANOVA) using Excel 2013 and SPSS 22.0 (SPSS, Inc., Chicago, Illinois), respectively. All the results are expressed as the mean ± standard deviation (x ± SD). ANOVA was used to determine significant intergroup differences. Fisher’s least significant difference (LSD) test was used to explore the inhibition of various indicators in different treatments and with different treatment durations (*p* = 0.05).

## 3 Results

### 3.1 Synergistic effect of *Bacillus tequilensis* combined with difenoconazole

#### 3.1.1 Synergistic inhibition of *Alternaria alternata* mycelia

Microbial chemical fungicide synergy test results: The synergistic antifungal activity of the microbial agent and chemical fungicide against *A. alternata* was significantly greater than that of *B. tequilensis* or difenoconazole alone. The antifungal activity of *B. tequilensis* combined with difenoconazole reached 90.77% (Table 2; Supplementary Figure 1).

**Table 2:** Inhibition of mycelia, spores, and PBS lesions by *B. tequilensis* combined with difenoconazole (%).

Treatment	Mycelial diameter (mm)	Mycelial inhibition rate (%)	Spore germination rate (%)	Germination inhibition rate (%)	Lesion area (mm^2^)	Lesion inhibition rate (%)
*B. tequilensis* + difenoconazole 1 × 10^5^ cfu/mL + 1 μg • mL^−1^	7.6 ± 0.0 c	90.77 ± 0.25 a	0.10 ± 0.02 d	99.90 ± 0.25 a	8.10 ± 0.40 c	99.53 ± 0.01 a
*B. tequilensis* 1 × 10^5^ cfu/mL	40.7 ± 0.3 b	50.77 ± 0.05 b	7.50 ± 0.20 c	92.17 ± 0.39 b	595.75 ± 8.80 b	65.23 ± 0.65 b
Difenoconazole 1 μg • mL^−1^	41.9 ± 0.3 b	49.23 ± 0.10 b	15.25 ± 0.50 bc	84.07 ± 0.42 c	765.10 ± 11.78 b	55.86 ± 0.89 b
Blank control	82.7 ± 0.7 a	–	95.75 ± 0.25 a	–	1733.33 ± 20.25 a	–

The data for the spore germination rate, germination inhibition rate, lesion area, and lesion inhibition rate are presented as the means ± SDs of the four corresponding replicates. The columns indicate significant differences at the *p* < 0.05 level according to the LSD test (the same applies below).

**Figure 1:**
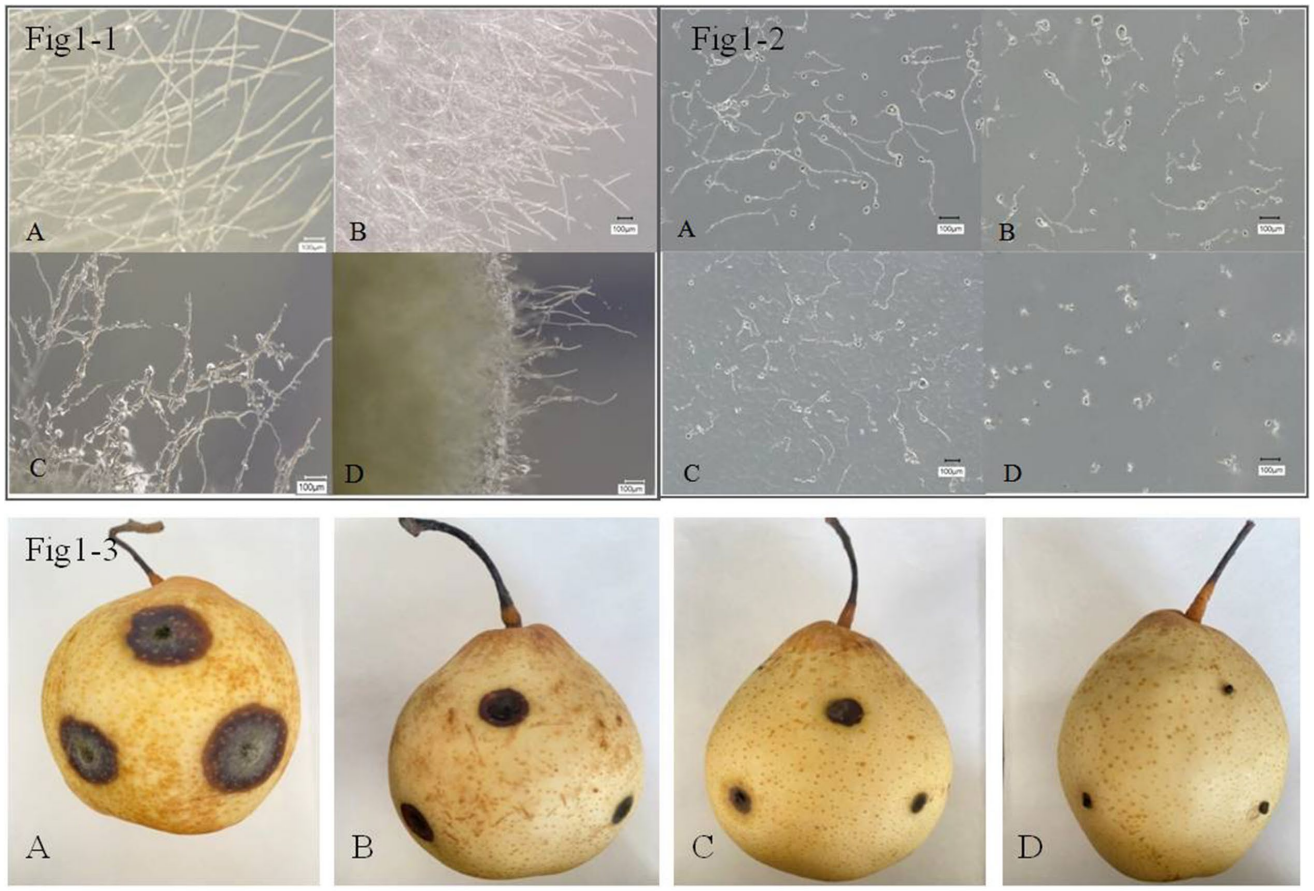
Inhibitory effect of *B. tequilensis* in combination with difenoconazole against *A. alternata* mycelia, spores and pear black spot *in vitro*. **(1-1-A, 1-2-A, 1-3-A)**: blank control; **(1-1-B, 1-2-B, 1-3-B)**: difenoconazole (1 μg • mL^1^); **(1-1-C, 1-2-C, 1-3-C)**: *B. tequilensis* (1 × 10 cfu/mL); **(1-1-D, 1-2-D, 1-3-D)**: *B. tequilensis* + difenoconazole (1 × 105 cfu/mL + 1 μg • mL).

Microscopic observations were conducted and showed that *B. tequilensis* and difenoconazole had synergistic antifungal effects against *A. alternata* (Figures 1-1). The blank control *A. alternata* had thicker, smoother, and more distinct branching hyphae. The mycelia treated with difenoconazole tended to exhibit thin and weak branching and aggregation, while the mycelia treated with *B. tequilensis* exhibited less branching, bending, and entanglement. However, the combination of *B. tequilensis* and difenoconazole shortened the hyphal length, resulting in unclear branching and entanglement. These findings indicate that the synergistic effect on *A. alternata* hyphae was more significant than that of the individual treatments.

#### 3.1.2 Synergistic inhibition of *Alternaria alternata* spore germination

**Figure 2:**
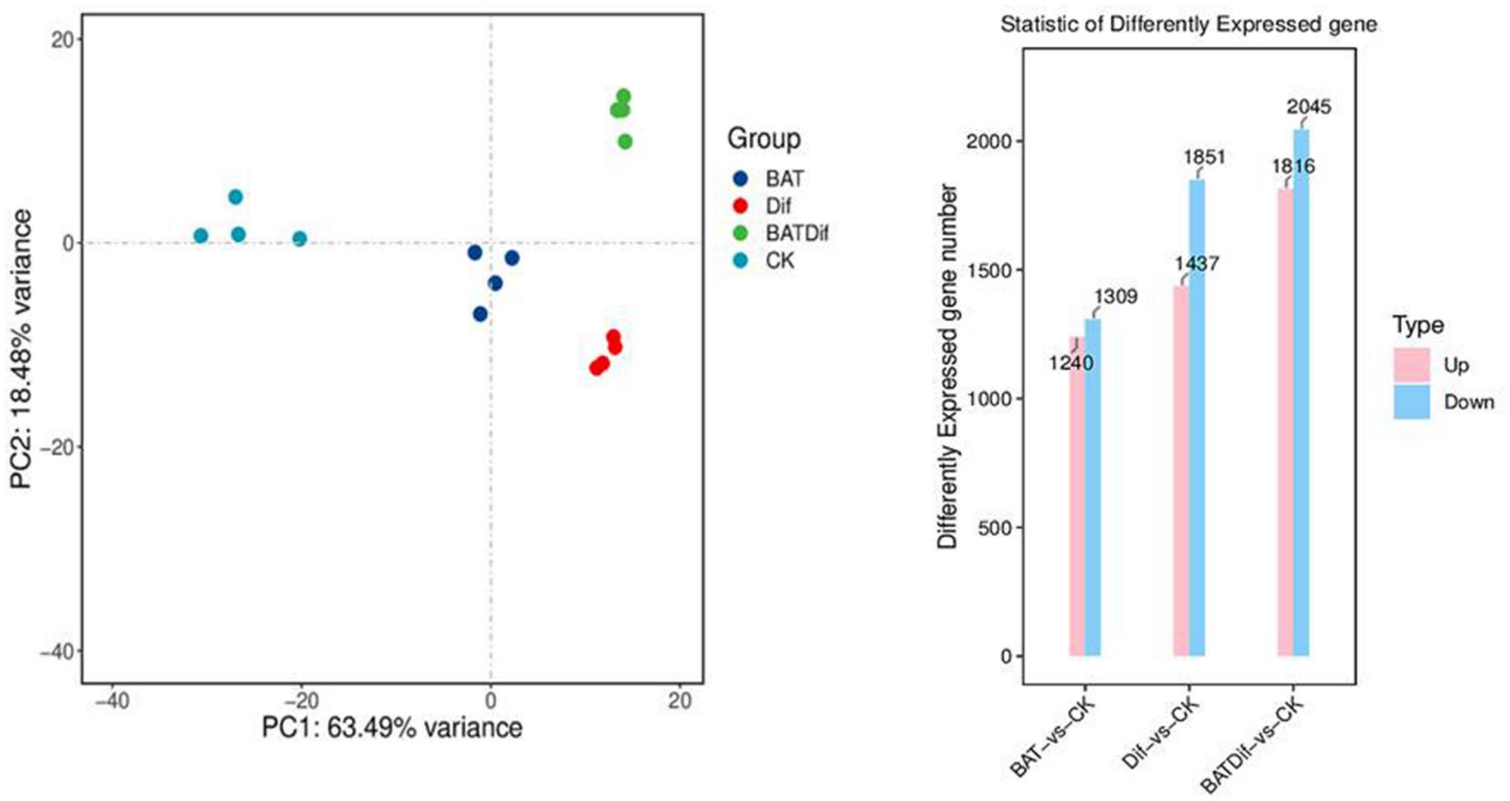
PCA and column chart analysis of RNA-Seq data to identify differential expression at the gene level in samples subjected to different treatments.

The rate of inhibition of *A. alternata* spore germination by *B. tequilensis* in combination with difenoconazole was as high as 99.90% (Table 2). Microscopic observation revealed that the blank control *A. alternata* had the highest spore germination rate and exhibited significant elongation of bud tubes. Similarly, compared with those of the blank control group, the germination rate of spores treated with difenoconazole decreased significantly, and the germinated bud tubes were broken. The germination rate of spores treated with *B. tequilensis* also decreased significantly, and the germ tubes were curved. However, the spore germination rate was the lowest for *B. tequilensis* combined with difenoconazole, and even the elongation of the germinated bud tubes was not significant (Figures 1-2).

#### 3.1.3 *In vitro* synergistic antifungal activity against PBS lesions

**Figure 3:**
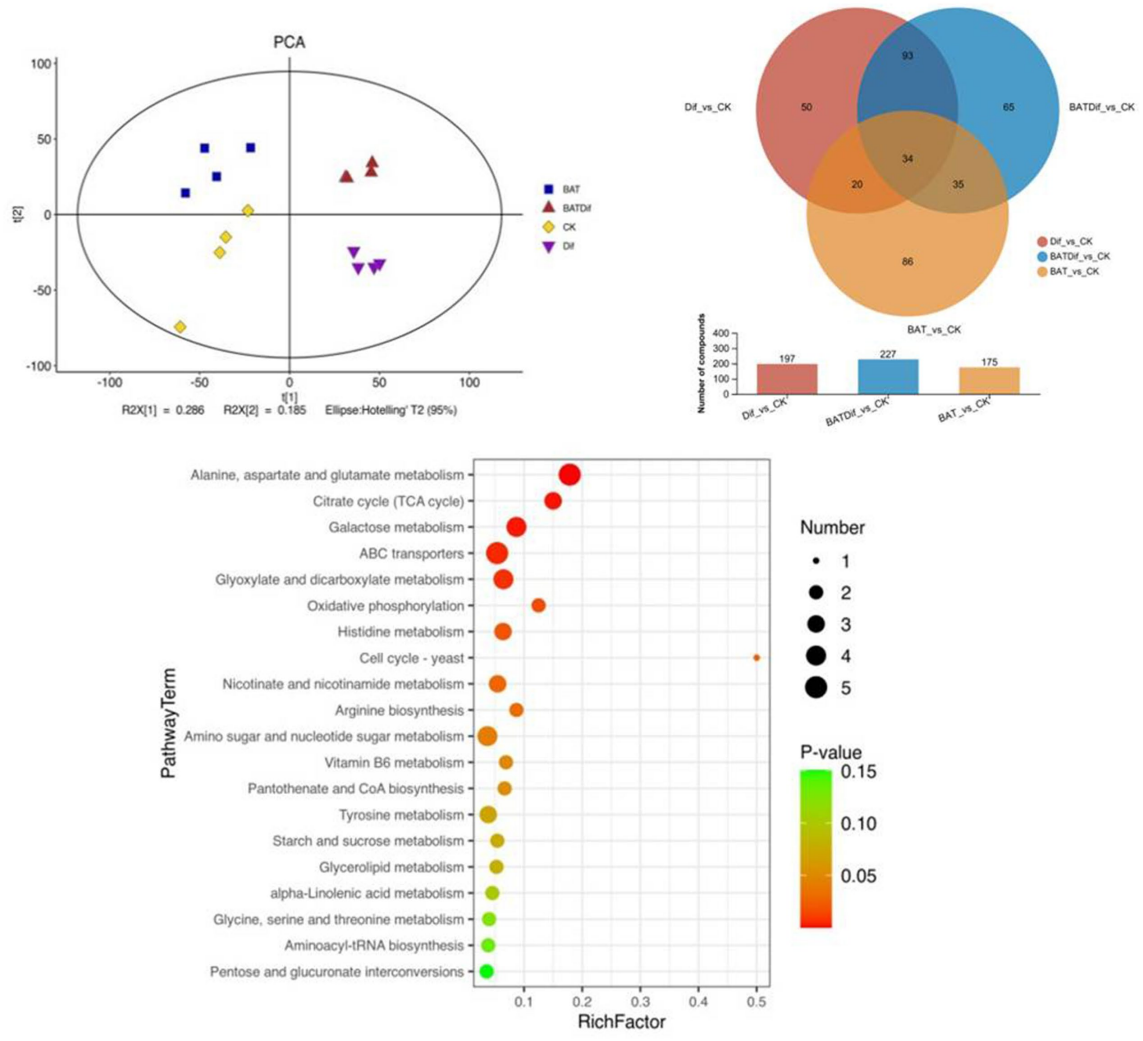
PCA, venn analysis, and KEGG enrichment analysis of significantly differentially abundant metabolites in samples subjected to different treatments.

The inhibition rate of *B. tequilensis* combined with difenoconazole against PBS formation was 99.53% (Table 2, Figures 1-3). Observations revealed that the lesions in the treatment with *B. tequilensis* combined with difenoconazole were significantly smaller than those in the individual treatments, and these lesions were significantly drier and did not expand.

#### 3.1.4 Synergistic effect on PBS in the field

To further examine the synergistic effect of *B. tequilensis* combined with difenoconazole, field tests were conducted. The PBS severity after treatment with *B. tequilensis* combined with difenoconazole was much lower than that in the individual treatment groups (Table 3). In 2020, 2021, and 2022, the control effects of *B. tequilensis* combined with difenoconazole against PBS on leaves were 95, 95, and 96%, respectively, with an average control effect of 95%. Similarly, the control effects on fruit were 98, 96, and 99%, with an average control effect of 98%. The synergistic effect of *B. tequilensis* combined with difenoconazole was significantly greater than that of *B. tequilensis* alone or difenoconazole alone.

**Table 3:** Synergistic effects of *B. tequilensis* combined with difenoconazole against PBS in the field.

Test year	Treatment	Investigation indicators			
		Leaves		Fruits	
		DI	CE (%)	IR	CE (%)
2020	*B. tequilensis* + difenoconazole 1 × 10^5^ cfu/mL + 1 μg • mL^−1^	1.70 ± 0.10	95 ± 0.1 a	0.49 ± 0.11	98 ± 0.1 a
	*B. tequilensis* 1 × 10^5^ cfu/mL	8.58 ± 0.40	74 ± 0.1 a	9.95 ± 0.74	64 ± 0.2 b
	Difenoconazole 1 μg • mL^−1^	13.27 ± 0.75	60 ± 0.2 b	11.00 ± 0.98	60 ± 0.2 b
	Blank control	33.40 ± 1.85	–	27.50 ± 2.45	–
2021	*B. tequilensis* + difenoconazole 1 × 10^5^ cfu/mL + 1 μg • mL^−1^	1.40 ± 0.08	95 ± 0.1 a	0.75 ± 0.22	96 ± 0.2 a
	*B. tequilensis* 1 × 10^5^ cfu/mL	7.95 ± 0.60	72 ± 0.2 b	7.35 ± 0.85	65 ± 0.4 b
	Difenoconazole 1 μg • mL^−1^	12.65 ± 1.05	55 ± 0.9 c	8.20 ± 1.34	61 ± 0.8 b
	Blank control	28.15 ± 2.24	–	20.96 ± 3.85	–
2022	*B. tequilensis* + difenoconazole 1 × 10^5^ cfu/mL + 1 μg • mL^−1^	1.57 ± 0.12	96 ± 0.2 a	0.27 ± 0.05	99 ± 0.3 a
	*B. tequilensis* 1 × 10^5^ cfu/mL	9.75 ± 0.80	74 ± 0.6 b	9.14 ± 0.27	71 ± 0.6 b
	Difenoconazole 1 μg • mL^−1^	14.25 ± 0.92	61 ± 0.8 b	13.62 ± 0.84	57 ± 0.9 c
	Blank control	36.98 ± 1.50a	–	31.50 ± 2.05 a	–

DI represents the disease index, IR represents the incidence rate, and CE represents the control effect. The data for DI, IR and CE are presented as the mean ± SD of four corresponding replicates.

### 3.2 Combined transcriptomic and metabolomic analysis of the synergistic effect

The mycelia of *A. alternata* treated with the combination of *B. tequilensis* and difenoconazole were cultivated for transcriptomic and metabolomic analysis, as were those from the corresponding individual treatment and blank control groups. The synergistic effect on *A. alternata* mycelia was more significant than that of the individual treatments (Supplementary Table 2; Figure 2). After completing the transcriptomic analysis of the 16 samples, which included the samples treated with the combination of *B. tequilensis* and difenoconazole and the corresponding individual treatment and blank control samples, a total of 104.06 Gb of clean data were obtained. The sequence homology rates ranged from 85.18 to 90.05%. The sequencing data have been submitted to the NCBI database (SRA access: PRJNA-962067).

PCA revealed that the difference in the transcriptome between the *B. tequilensis* combination treatment group and difenoconazole treatment group was greater than that between the two treatment groups. The number of DEGs in *A. alternata* treated with this combination was greater than that in the corresponding individual treatment groups. A total of 3,861 genes were differentially expressed between the blank control group, including 1816 upregulated and 2045 downregulated genes (Figure 2). The relationship between these DEGs and those identified after treatment with the combination of *B. tequilensis* and difenoconazole will be analyzed in a follow-up study. The main signaling pathways annotated by KEGG analysis included glycolysis/glycogenesis, oxidoreductase activity/oxidative phosphorylation, the pentose phosphate pathway/conversion of pentose and uronic acid, the citric acid cycle (TCA cycle), acetyl-CoA synthase-like/acetate CoA ligase/acyl CoA dehydrogenase family/CoA hydratase mitochondrial precursor/CoA transferase family, steroid biosynthesis ATP citrate synthase/ATP-dependent DNA helicase/ADP ribose pyrophosphatase/ADP ribosylation factor family AMP deaminase, NAD (P)-binding protein/NADPH dehydrogenase, the cell cycle, cytochrome P450, ABC transport, membrane components, the MAPK signaling pathway, autophagy, MFS transport, and metabolism of various amino acids and purines (Table 4).

**Table 4:** Gene numbers of typical enriched KEGG signaling pathways and annotations.

Main pathway description	Number of genes enriched	Number of annotated genes
Glycolysis/Gluconeogenesis	26	120
oxidoreductase activity/Oxidative phosphorylation	22	64
Pentose phosphate pathway/Pentose and glucuronate interconversions	18	81
Valine, leucine and isoleucine biosynthesis|Pantothenate and CoA biosynthesis	12	66
Ubiquinone and other terpenoid-quinone biosynthesis	12	70
Cell cycle	12	134
integral component of membrane	10	42
Glycine, serine and threonine metabolism	9	57
Fatty acid biosynthesis/degradation	7	49
MAPK signaling pathway	8	119
Steroid biosynthesis	7	43
Citrate cycle (TCA cycle)	6	32
Autophagy	4	35
Alanine, aspartate, and glutamate metabolism	3	15
Amino sugar and nucleotide sugar metabolism	3	16
Endocytosis	3	21
Arginine biosynthesis	2	5
Glyoxylate and dicarboxylate metabolism	2	13
NAD(P)-binding protein/NADPH dehydrogenase	115	385
MFS general substrate transporter	43	143
ATP-citrate synthase/ATP-dependent DNA helicase/ADP-ribose pyrophosphatase/ADP-ribosylation factor family AMP deaminase	31	286
cytochrome P_450_	30	83
acetyl-CoA synthetase-like/acetate--CoA ligase/acyl-CoA dehydrogenase family/CoA hydratase mitochondrial precursor/CoA-transferase family	28	49
ABC transporter	12	39

The metabolomic analysis of four replicates of *B. tequilensis* in combination with difenoconazole was completed, as well as that of the corresponding individual treatment and blank control treatments. The PCA results showed that the difference in the metabolome of *A. alternata* treated with the combination of *B. tequilensis* and difenoconazole was greater than that in the individual treatment groups (Figure 3). The synergistic effect of the *B. tequilensis* and difenoconazole combination on metabolite levels in *A. alternata* resulted in significant differences in the abundances of 227 metabolites, including 103 upregulated and 124 downregulated metabolites. The number of differentially abundant metabolites in *A. alternata* treated with this combination was greater than that in the corresponding individual treatment groups (Figure 3). KEGG enrichment analysis of differentially abundant metabolites in *A. alternata* treated with the combination of *B. tequilensis* and difenoconazole revealed that pathways involved in ABC transmembrane transport, sugar metabolism, the tricarboxylic acid (TCA) cycle, oxidative phosphorylation, the conversion of pentose and glucuronic acid, coenzyme A biosynthesis, the metabolism of various amino acids, ester metabolism, the biosynthesis of aminoacyl tRNA and the conversion of pentose and glucuronic acid were highly enriched (Figure 3).

**Figure 4:**
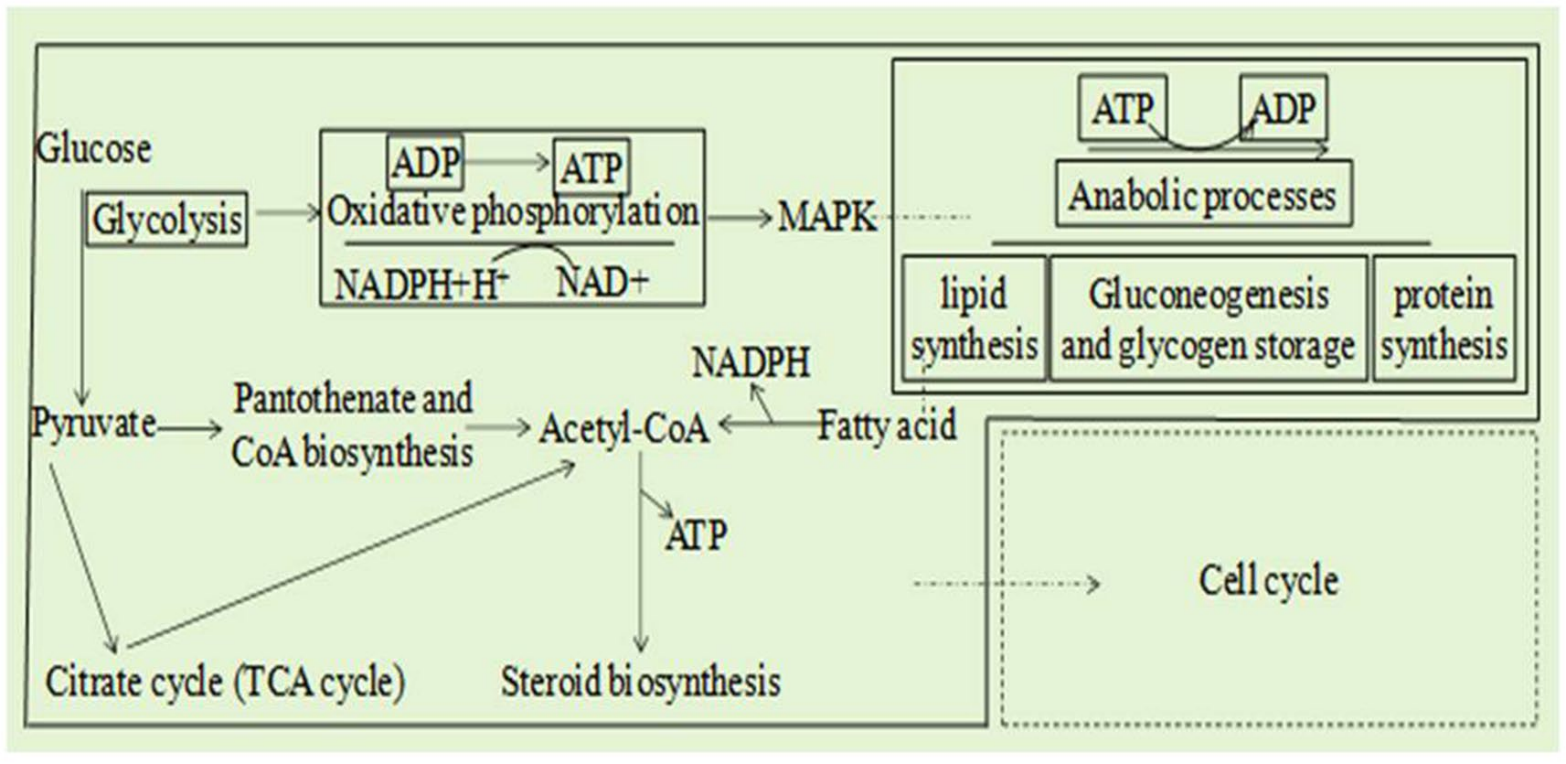
Eight integrated transcriptomic and metabolomic pathway diagrams and a core regulatory network model of the effects of *Bacillus tequilensis* in combination with difenoconazole against *Alternaria alternata.*

Combined analysis of the transcriptome and metabolome of *A. alternata* treated with *B. tequilensis* in combination with difenoconazole indicated that the main synergistic effects involved eight pathways (Figure 4): glycolysis/gluconeogenesis, the TCA cycle, coenzyme A biosynthesis, oxidative phosphorylation, fatty acid degradation, the sterol synthesis pathway, the MAPK signaling pathway and the cell cycle. These results indicate that the glycolytic pathway affects the production of pathogen pyruvate, leading to abnormal decarboxylation and the production of acetyl-CoA. The TCA cycle is disrupted to a certain extent, and protons cannot be completely transferred to the coenzyme nicotinamide adenine. NADH^+^H^+^ cannot be further oxidized to NAD in the respiratory chain. Oxidative phosphorylation inhibition results in ADP not being converted to ATP, and reduced ATP does not couple with the electron transfer chain. Sterols cannot be synthesized normally, resulting in the inability to construct lipids and complete oxidation of lipids to produce acetyl-CoA, further affecting the production of ATP. The metabolic transition from glycolysis to oxidative phosphorylation and the disruption of lipid synthesis pathways inhibit normal MAPK signal transduction. The combined effects of the above factors collectively affect the cell cycle. A joint analysis diagram was constructed for the eight pathways underlying the synergistic effect of *B. tequilensis* and difenoconazole against *A. alternata*, and it was determined that the effects on these pathways were mutually influential (Figure 4).

### 3.3 Verification of the genes and metabolic components in major signaling pathways

#### 3.3.1 Verification of the main genes involved in the synergistic effect

**Figure 5:**
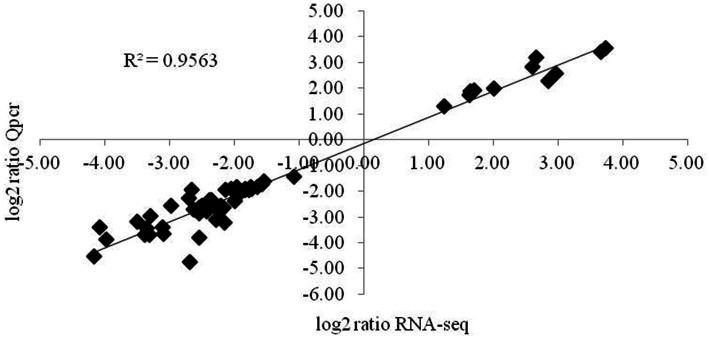
Validation of the main differentially expressed genes by RT-qPCR. The changes in the expression of the main differentially expressed genes determined by RT-qPCR were consistent with the RNA-seq results. The data are presented as 2-4Act values (mean ± SE).

The expression levels of genes associated with the synthesis of glucose and citric acid, oxidative phosphorylation, the mutual conversion of pentose and glucuronic acid, oxidative cytochrome P_450_, cell membrane components, lipid synthesis and degradation, sterol synthesis, acetyl-CoA synthesis, MAPK signal transduction, cell circulation, MFS transport, and ABC transport and of the intracellular pyridine nucleotides involved in oxidative phosphorylation and lipid synthesis, adenine nucleotides providing energy, various amino acid synthesis genes, ubiquinone biosynthesis genes, and autophagy- and secretion-related genes involved in various processes were validated by fluorescence quantitative PCR. The correlation coefficient of the 57 key genes validated was 0.9563 (Figure 5), and the expression levels of these main target genes were basically consistent.

#### 3.3.2 Verification of the metabolic components involved in the synergistic effect

The ECAR and OCR are key indicators of glycolysis, mitochondrial respiration, and the ATP production rate. The ECAR and OCR of *A. alternata* treated with the combination of *B. tequilensis* and difenoconazole decreased significantly, and the inhibitory effect was significantly greater than that of the corresponding single treatments (Table 5).

**Table 5:** Effects of *B. tequilensis* in combination with difenoconazole on ECAR, OCR and intracellular pyridine nucleotides in *A. alternata.*

Treatment	ECAR				OCR				Intracellular pyridine nucleotides					
	30 min	60 min	90 min	120 min	30 min	60 min	90 min	120 min	NADH	NAD+	NADH/ NAD+	NADPH	NADP+	NADPH/NADP+
*B. tequilensis* + difenoconazole 1 × 10^5^ cfu/mL +1 μg • mL^−1^	12.03 ± 0.45 b	5.02 ± 0.05c	3.40 ± 0.04 c	1.02 ± 0.12 c	12.30 ± 0.05 b	9.42 ± 0.81c	6.03 ± 0.50 c	4.16 ± 0.15 c	3.05 ± 0.23 a	3.20 ± 0.15 b	0.90 ± 0.02 a	0.05 ± 0.01 c	2.90 ± 0.11 a	0.02 ± 0.01 b
*B. tequilensis* 1 × 10^5^ cfu/mL	22.11 ± 0.47 a	8.13 ± 0.13 b	5.05 ± 0.10 b	4.15 ± 0.20 b	35.36 ± 0.77 b	26.95 ± 2.13 b	24.38 ± 1.18 b	22.25 ± 2.11 b	2.84 ± 0.25 ab	6.82 ± 0.14 a	0.40 ± 0.02 b	0.10 ± 0.01 b	2.52 ± 0.05 ab	0.04 ± 0.01 b
Difenoconazole 1 μg • mL^−1^	18.04 ± 0.75 b	8.25 ± 0.30 b	6.13 ± 0.50 b	3.20 ± 0.32 b	30.47 ± 1.15 b	24.05 ± 3.22 b	20.19 ± 2.04 b	16.09 ± 3.25 b	2.50 ± 0.12 ab	4.22 ± 0.18 ab	0.65 ± 0.02 ab	0.12 ± 0.01 b	2.68 ± 0.06 ab	0.03 ± 0.01 b
Blank control	24.12 ± 1.00 a	24.31 ± 0.90 a	26.06 ± 0.82 a	26.34 ± 0.41 a	108.10 ± 2.00 a	270.46 ± 5.18 a	260.88 ± 4.12 a	255.60 ± 6.01 a	1.85 ± 0.12 b	7.80 ± 0.25 a	0.20 ± 0.02 c	1.22 ± 0.01 a	1.01 ± 0.04 b	1.01 ± 0.05 a

The ECAR, OCR, and intracellular pyridine nucleotides content data are presented as the means ± SDs of four replicates.

Citrate synthase in the TCA cycle is regulated mainly through feedback inhibition by citric acid. Compared with those in the blank control, the citric acid content and relative expression of citric acid synthase in *A. alternata* after the synergistic effect of *B. tequilensis* combined with difenoconazole significantly decreased by more than 81%, which was significantly greater than the decrease observed in the corresponding individual treatments (28 and 56%, respectively) (Table 6).

**Table 6:** Effects of *B. tequilensis* in combination with difenoconazole on citric acid, citrate synthase, CoA, and intracellular adenine nucleotides in *A. alternata.*

Treatment	Citric acid		Citrate synthase		CoA content (nmol /mg)	Inhibition rate (%)	Sterol content (μg/g)	Inhibition rate (%)	Intracellular adenine nucleotides (μmol) (g DCW)^−1^			Relative inhibition (%)		
	Content (μmol /g)	Inhibition rate (%)	Relative expression	Inhibition rate (%)					ATP	ADP	AMP	ATP	ADP	AMP
*B. tequilensis* + difenoconazole 1 × 10^5^ cfu/mL +1 μg • mL^−1^	3.41 ± 0.03 c	81.37 ± 0.02 a	0.29 ± 0.08 c	84.24 ± 0.04 a	7.10 ± 0.08 c	80.14 ± 0.06 a	5.77 ± 0.16 c	92.62 ± 0.05 a	0.55 ± 0.03 c	3.18 ± 0.15 a	0.90 ± 0.04 a	87.96 ± 0.02 a	95.09 ± 0.11 a	57.89 ± 0.03 a
*B. tequilensis* 1 × 10^5^ cfu/mL	8.62 ± 0.09 b	52.90 ± 0.07 b	0.85 ± 0.17 b	53.80 ± 0.10 b	22.82 ± 0.39 b	36.17 ± 0.25 b	55.22 ± 0.80 ab	29.35 ± 0.35 c	3.04 ± 0.19 ab	2.32 ± 0.14 ab	0.68 ± 0.05 b	33.48 ± 0.08 c	42.33 ± 0.09 b	19.30 ± 0.03 c
Difenoconazole 1 μg • mL^−1^	13.68 ± 0.14 ab	25.25 ± 0.11 c	1.42 ± 0.22 ab	22.83 ± 0.15 c	21.35 ± 0.44 b	40.28 ± 0.31 b	31.65 ± 0.66 b	59.51 ± 0.41 b	2.17 ± 0.08 b	1.95 ± 0.22 b	0.75 ± 0.02 ab	52.52 ± 0.03 b	19.63 ± 0.10 c	31.58 ± 0.03 b
Blank control	18.31 ± 0.20 a	–	1.85 ± 0.16 a	–	35.75 ± 0.95 a	–	78.16 ± 0.92 a	–	4.57 ± 0.21 a	1.63 ± 0.09 b	0.57 ± 0.10 c	–	–	–

The citric acid, citrate synthase, CoA, and adenine nucleotide content data are presented as the means ± SDs of four replicates.

The synthesis of all sterols in eukaryotes begins with acetyl-CoA (CoA). The reduction in CoA content resulting from the synergistic effect of *B. tequilensis* and difenoconazole reached 80.14%, which was significantly greater than that caused by the corresponding individual treatments (43 and 39%, respectively) (Table 6).

Intracellular pyridine nucleotides are the metabolic basis of energy and are responsible for cellular respiration and partial biosynthetic reactions. The synergistic effect of *B. tequilensis* and difenoconazole increased the intracellular NADH/NAD^+^ ratio, and the increase in NADH inhibited this oxidation. A decrease in the NADPH/NADP^+^ ratio and the level of NADPH affected the normal synthesis of substances required by pathogens (Table 5).

Adenine nucleotides are important for energy production by pathogenic bacteria and regulate normal functions via pathways such as glycolysis, the TCA cycle, the electron transfer system, oxidative phosphorylation, and AMPK. The synergistic effect of *B. tequilensis* and difenoconazole reduced the ATP content by 87.96%, while it increased the ADP and AMP content by 95.09 and 57.89%, respectively, which were significantly greater than the levels in the corresponding individual treatments (54, 35, 53, 75, 38, and 26%, respectively) (Table 6).

Sterols participate in various signaling pathways and can bind to receptors on the cell membrane, triggering intracellular signal transduction and thereby affecting cell function and metabolism. The synergistic effect of *B. tequilensis* and difenoconazole on the biosynthesis of pathogenic sterols was antifungal, with an inhibitory effect of up to 92.62% on sterol synthesis, which was significantly greater than the effects of the corresponding individual treatments (63 and 33%, respectively) (Table 6).

## 4 Discussion

At present, biocontrol agents are gradually being applied for disease prevention and control, and the synergistic effect of biocontrol agents and chemical agents can effectively antagonize target pathogens. Currently, most biocontrol agents cannot be used alone to fully control severe and prevalent plant diseases (Liu et al., 2015). The alternating or combined use of biological control agents and chemical agents inevitably results in the residual presence of chemical agents in the environment. Therefore, studying the synergistic effects and mechanisms of biocontrol agents and chemical agents has important practical significance for identifying specific and effective ways to correctly select and reduce the use of chemical agents and providing technical support for pear cultivation.

The combined use of *Bacillus* spp. and chemical fungicides has potential applications in the prevention and control of plant diseases. Research has shown that the combined use of *B. tequilensis* 2-2a and difenoconazole has good inhibitory effects on mycelia, spore germination, *in vitro* PBS lesions, and PBS in the field. The main fungicides currently used to prevent and control PBS are sterol 14 α-demethylation inhibitors (DMIs), such as difenoconazole and tebuconazole; these fungicides have been rapidly developed and are widely used due to their wide antifungal spectrum. Plant pathogens such as *Fusarium graminearum*, *Erysiphe graminis*, *Penicillium digitatum*, and *Mycosphaerella gramicola* have all been reported to develop resistance to DMI fungicides in the field (Stammler et al., 2008; Yan et al., 2009; Liu et al., 2011; Sánchez-Torres and Tuset, 2011). In most cases, microbial-chemical fungicide synergy can effectively control resistant pathogens by simultaneously targeting multiple defense mechanisms. The synergistic effect of *Bacillus* spp. and the steroid inhibitor mefentrifluconazole on pathogenic fungi at different stages significantly inhibited the growth of hyphae and spores of the rice blast pathogen (Chen et al., 2020). Liu et al. (2023) reported that *Bacillus* spp. and the sterol inhibitor tebuconazole exert their inhibitory effects by disrupting the biofilms of pathogenic fungi. These results are consistent with the results showing the synergistic effect of *B. tequilensis* 2-2a and difenoconazole on pathogenic fungi at different stages. The above explanation shows that *B. tequilensis* exhibits good synergy with chemical fungicides.

Elucidating the mechanism underlying the synergistic effect of *B. tequilensis* with difenoconazole can provide a scientific basis for guiding the scientific application of microbial and chemical fungicide combinations. There are two main pathways underlying the synergism between *B. tequilensis* and difenoconazole. One type includes glycolysis, oxidative phosphorylation, and MAPK signal transduction, while the other includes glycolysis, the TCA cycle, coenzyme A biosynthesis, sterol synthesis, and fatty acid degradation. Both types of pathways jointly affect the cell cycle. The main functions of the key genes and metabolites affected in these pathways include glucose synthesis and oxidative respiration as well as citric acid synthesis, acetyl-CoA synthesis, and sterol synthesis, all of which involve substance transport, metabolism of intracellular pyridine nucleotides (NAD^+^, NADH, NADP^+^, and NADPH), and conversion of adenine nucleotides (ATP, ADP, and AMP). There are correlations among the above pathways.

The pathways underlying the synergistic effects of *B. tequilensis* and difenoconazole are complex. The glycolytic pathway is at the centre of the entire control system. The ECAR of *A. alternata* decreased significantly upon treatment with the combination of *B. tequilensis* and difenoconazole. Li et al. (2023) reported that changes in the expression of genes involved in glycolytic pathways can affect glucose metabolic utilization. The citric acid content and relative expression of citric acid synthase in *A. alternata* decreased significantly after treatment with *B. tequilensis* combined with difenoconazole. The TCA cycle connects the metabolism of amino acids or fat and glycolysis, participating in the AMPK pathway (Tao et al., 2017). The inhibition of the TCA cycle weakens respiration and lowers the contents of acetyl-CoA and ATP (Nordström, 1963). The reduction in CoA content resulted from the synergistic effect of *B. tequilensis* and difenoconazole. A reduction in the transmembrane transport of ATP can hinder the input and output of substances involved in cellular processes. The synergistic effect of *B. tequilensis* and difenoconazole reduced the content of ATP and increased the contents of ADP and AMP; ADP and AMP could not be rapidly converted to ATP. NADH, NADPH, and ATP are affected, as are oxidative phosphorylation and glycolytic pathways (Behrends et al., 2019). The synergistic effect of *B. tequilensis* and difenoconazole increased the level of NADH and decreased the level of NADPH, which affected oxidation and the normal synthesis of substances required by pathogens. *B. subtilis* HSY21 is also a potential antibacterial target for inhibiting sterol synthesis in *F. oxysporum* (Han et al., 2023). The combination of *B. tequilensis* 2-2a and difenoconazole could inhibit sterol synthesis, which was consistent with previous results. Moreover, the results of the present study revealed that the ABC and MFS transporter protein genes are involved in the main molecular mechanisms by which pathogens resist sterol inhibitors. The ABC-binding cassette transporter or MFS cotransporter superfamily comprises efflux pumps in fungi (Sun et al., 2022). Under the action of pesticides, the low expression of genes encoding efflux pumps inhibits this efflux function, reducing the rate of drug efflux from the cell (Omrane et al., 2015). The synergistic inhibition by *B. tequilensis* 2-2a and difenoconazole led to a decrease in drug excretion, increased fungicide accumulation in the pathogen, and enhanced the destructive effect on the pathogen. It is thus clear that the pathways underlying the synergistic effects of *B. tequilensis* and difenoconazole are consistent with the above-reported results.

The use of chemical agents can enable biocontrol bacteria to better exert their biocontrol effects, and the utilization of biocontrol bacteria can effectively reduce the amount of chemical agents used. The synergistic effect of these two agents can enhance the destructive effect against pathogens. The construction of a complete molecular target-based antibacterial regulatory system, the targeting of key inhibitory sites on pathogenic fungi, and the study of the synergistic mechanism of action of *B. tequilensis* with difenoconazole have laid the foundation for the targeted application of this combination in disease control.

## 5 Conclusion

The synergistic effects of *B. tequilensis* 2-2a and difenoconazole were evaluated by testing the antifungal effects on hyphae, spores, and detached-fruit lesions *in vitro*; testing the field control effects; transcriptome and metabolome profiling; qRT–PCR-based key target gene expression analysis; and metabolome validation. The synergistic mechanism of action can be described as follows: *B. tequilensis* 2-2a exerts antifungal effects by inhibiting significant DEGs and metabolic components in the key cycling pathway of *A. alternata*. These results indicate that the combination of *B. tequilensis* 2-2a and difenoconazole can be used to prevent and treat PBS caused by *A. alternata*.

## Data availability statement

The datasets presented in this study can be found in online repositories. The names of the repository/repositories and accession number(s) can be found in the article/Supplementary material.

## Ethics statement

The participants provided written informed consent to participate in this study. Written informed consent was obtained from the individual(s) for the publication of any potentially identifiable images or data included in this article.

## Author contributions

QB: Writing – review & editing, Writing – original draft, Visualization, Validation, Supervision, Software, Resources, Project administration, Methodology, Investigation, Funding acquisition, Formal analysis, Data curation, Conceptualization. FL: Writing – review & editing, Supervision, Resources, Formal analysis, Data curation, Conceptualization. JW: Writing – review & editing, Resources, Investigation, Data curation. XL: Writing – review & editing, Visualization, Formal analysis. XH: Writing – review & editing, Software, Formal analysis. WW: Writing – review & editing, Validation, Methodology. JZ: Writing – review & editing, Project administration.
