# Supplementary material for: Combination of Bacillus tequilensis with difenoconazole to control pear black spot and the related synergistic mechanism
Source: Front Microbiol. 2024 Jun 4;15:1405039. doi: 10.3389/fmicb.2024.1405039 (PMC11183105; doi:10.3389/fmicb.2024.1405039)
Supplement: Supplementary file 1 [file Data_Sheet_1.docx]

Supplementary Material

## 1. Supplementary Figures

**Fig. 1.** Antibacterial activities of *B. tequilensis* in combination with difenoconazole against *A. alternate* mycelia

Figure 1-A: *A. alternata* was inoculated and cultured on PDA plates with different treatments for 7 days; figure 1-B: *A. alternata* was inoculated and cultured on PDA plates with different treatments for 10 days. The order from left to right in each figure is as follows: blank control, difenoconazole (1 μg•mL^-1^), *B. tequilensis* (1×10^5^ cfu/mL), and *B. tequilensis* + difenoconazole (1×10^5^ cfu/mL+1 μg•mL^-1^).


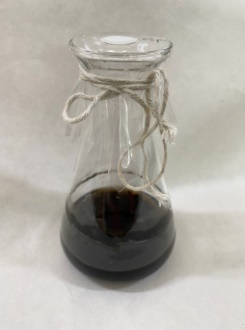

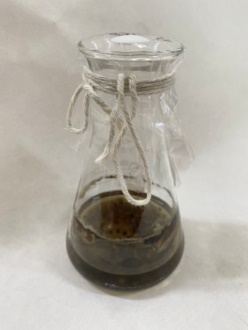

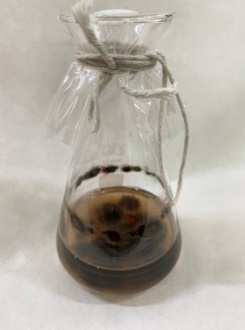

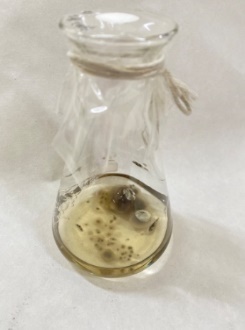

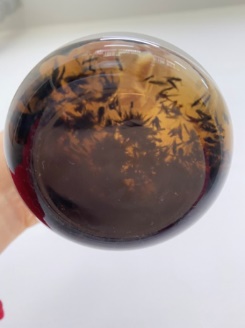

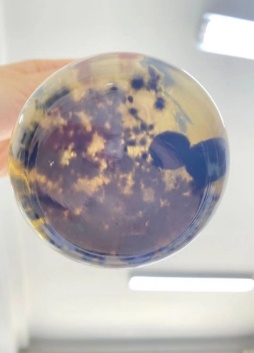

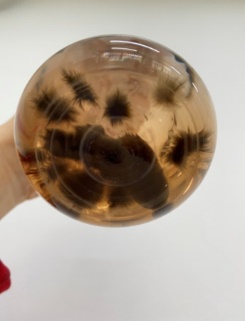

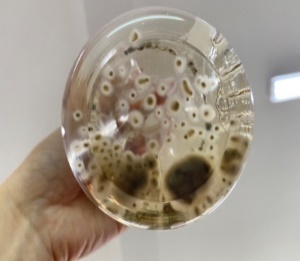


A

B

C

D

E

F

G

H

**Fig. 2.** Changes in the mycelial dry weight of *A. alternata* treated with *B. tequilensis* combined with difenoconazole

Upper figures 2-A to 2-D: Overall frontal view of shake-cultured *A. alternata* under different treatments; lower figures 2-E to 2-H: Partial bottom view of shake-cultured *A. alternata* under different treatments. The order from figure A to figure D and figure E to figure H in each row is as follows: blank control, difenoconazole (1 μg•mL^-1^), *B. tequilensis* (1×10^5^ cfu/mL), and *B. tequilensis* + difenoconazole (1×10^5^ cfu/mL+1 μg•mL^-1^).

## 2. Supplementary Tables

**Table 1.** List of qRT‒PCR primers used for fluorescence quantification

| **Gene name** | **Gene function description** | **Forwards primer (5’-3’)** | **Reverse primer (5’-3’)** |
| --- | --- | --- | --- |
| CC77DRAFT_1004828  (down) | Glycolysis/Gluconeogenesis | TGTTGGATCAGCTGTGGGAT | ACAACTCTGCCCTGTAGCTT |
| CC77DRAFT_1016116  (down) | Glycolysis/Gluconeogenesis | ATACGTCACTGCTGTTCCCA | ATGGCTACTTTGCTCTGGGT |
| CC77DRAFT_636933  (up) | Glycolysis/Gluconeogenesis | AGCTTCGCGATGACATGTTC | ACCTGCTCGCTAAACTCCTT |
| CC77DRAFT_1005031  (down) | Citrate cycle (TCA cycle) | TTCCCCAAAGATCTCCACCC | GCAGCAATGGTAGGCAGTTT |
| CC77DRAFT_250650  (down) | Citrate cycle (TCA cycle) | TGTACCTCACCATCCACACC | GCTGAGGTCGTTGCCAATAG |
| CC77DRAFT_1024999  (down) | Oxidative phosphorylation | TACTGAGCACTGGGGAAAGG | CTGTGTCTTTCTCCATCGCG |
| CC77DRAFT_1022516  (down) | Oxidative phosphorylation | CTTGCAGCAGAATACGGTCC | TGCCTCCTTACTTGCCATGA |
| CC77DRAFT_1081377  (down) | Pentose and glucuronate interconversions | CTACTCGGAGTGGAAGGGTC | ATGGTGGAGTCGGTCAGTTT |
| CC77DRAFT_1063881  (up) | Pentose and glucuronate interconversions | CAGAGGGGTTGTCAAGGCTA | ACCGGGCTTGAGAGTTGTAA |
| CC77DRAFT_948756 (down) | cytochrome P450 | ATGGCTTCTAAGGATCCGGG | GACTGTGTTGGCTAGGAGGT |
| CC77DRAFT_967093 (down) | cytochrome P450 | TCGATAACCCAGACTCACCG | GCTTGATCGATCGCTTGGTT |
| CC77DRAFT_1010772 (down) | cytochrome P450 | ACGCCGATTACTTCCCTCAA | CACCGTCAGCTCAACATACG |
| CC77DRAFT_1018362  (down) | integral component of membrane | ATGGTGAAGACTGCGGAGAA | AGTGGCATTGAAAGCGTTGT |
| CC77DRAFT_1013694  (down) | integral component of membrane | CGTACCCGCCGAAAATGTAG | TGGCCAGTGGATCTTTAGCA |
| CC77DRAFT_1016399  (up) | integral component of membrane | TACCTTCCTCCTCGACCAGA | CCATAGAACGGCGGAAAGTG |
| CC77DRAFT_928877  (down) | Fatty acid biosynthesis | GTGTCTTTGCTAGCTACGCC | ACCAGAAACCATACCGACGT |
| CC77DRAFT_265112  (down) | Fatty acid degradation | CCAACCCATCCTAACGACCT | TGGAGCCAAAGGTTACGTCT |
| CC77DRAFT_261211  (down) | Biosynthesis of unsaturated fatty acids | TGTCAACTCAGTCTCGGCTT | TGGTGTAGTCGGGGATAACG |
| CC77DRAFT_990455 (down) | Steroid biosynthesis | TTCGACCCTCTATACGCCAC | GCGTCCATTGGCTCATGTAG |
| CC77DRAFT_76456 (down) | Steroid biosynthesis | CCGATATCCTGGCCATCACT | AAGGAGATGACCATGCACCA |
| CC77DRAFT_1015801 (up) | Steroid biosynthesis | CTTGGTCTACATTGTCGCCG | CATCACTCCCATCAGCTTGC |
| CC77DRAFT_947345 (down) | acetyl-CoA synthetase-like protein | CATTGTCTACGCTCCACTGC | CACCCTTTTGCCCTCTTTCC |
| CC77DRAFT_1097638  (down) | acyl-CoA dehydrogenase family | CATTGTCTACGCTCCACTGC | CACCCTTTTGCCCTCTTTCC |
| CC77DRAFT_1057398  (down) | acyl-CoA N-acyltransferase | TACCGTGCTATCCGTCCAAG | TTCTTCCTTCGTTGCGCAAA |
| CC77DRAFT_1030172  (down) | MAPK signalling pathway | ACCCAAGAGCCAGATCGAAA | GATCCTACAACGGCGACAAC |
| CC77DRAFT_946700  (down) | MAPK signalling pathway | GCAAGAAGTTTGGAGGCACA | CGGTTTCCAGGTTCACGTTT |
| CC77DRAFT_156510  (down) | MAPK signalling pathway | ACACAGCCAATACCCCATGA | CCTTTGAAGCCCTTGGTGAC |
| CC77DRAFT_1098683  (down) | Cell cycle | CTTGAAGATGCAGACGAGGC | GTTAGTGCTGTGCTTTGCCT |
| CC77DRAFT_994577  (down) | Cell cycle | CCGACTCTATCTGGCATCGT | GAGGTGACCAATGTGCAGTG |
| CC77DRAFT_985514 (down) | MFS general substrate transporter | CCATCAGCCGTATCCTGGAT | TACCGTTCCCCAGTCCATTC |
| CC77DRAFT_590037 (down) | MFS general substrate transporter | AACTCGGGTCTTGGGTAGTG | GCATGCCAGAATCTCCAGTG |
| CC77DRAFT_213884 (down) | MFS general substrate transporter | TTTTCCAAGCCTCACAAGCC | TTGGCGAAAAGATGGTGGTG |
| CC77DRAFT_333965 (down) | MFS general substrate transporter | ACCCTTCATTGGAGGCTTCA | GCTTTGCGTGTATACCCCAG |
| CC77DRAFT_925715 (up) | MFS general substrate transporter | GAACCCTTTCGATTGGTCCG | GTCGTAAAGGTCGTGATGCC |
| CC77DRAFT_1039035(down) | ABC transporter | TGCACTGACACCATCTCCAT | TGACAACCATGCCACCAATG |
| CC77DRAFT_364501(down) | ABC transporter | ACTGACTGTCGACGACCTTT | ACATAGTCTGGGCGGAACAA |
| CC77DRAFT_924493(up) | ABC transporter | CTTACTCGAGCTTGCGTGAC | TCCGAAGTAGATCTGGCGAC |
| CC77DRAFT_1000211 (up) | NAD(P)-binding protein | AATGGGGATGTCTGCTTCGA | ACAACACCAATTTCGACCGG |
| CC77DRAFT_52654 (up) | NAD(P)-binding protein | TACTCTCGTGTCAAGGCTGG | CTAACTGCTGCGACTCCAAC |
| CC77DRAFT_1024752 (down) | NADPH dehydrogenase | CTCGCAAATGGAGCCTCTTC | CCAGCCGTTCATCTCCTTTG |
| CC77DRAFT_1027108 (up) | FAD/NAD(P)-binding domain-containing protein | ACGACGGCACATTCATTCAG | ATTGTCAAGGATAACGCCGC |
| CC77DRAFT_947590 (down) | NADPH oxidase | CACCACAATCAGCAAACCCA | TCATTGGGGCAGGAAGTGAT |
| CC77DRAFT_486143  (up) | AMP deaminase | CACACACGTTCACCACTCAG | CAGAGTCAGCAGATTGTCGC |
| CC77DRAFT_942416  (down) | ATP-dependent RNA helicase | TCATTTTCGTCAGGACCCGA | TTGATGACCATGGTGACGGA |
| CC77DRAFT_986135  (down) | ATP-citrate synthase-like protein | TTGGTCTCATCCGAAAGCCT | GAAACGGCTAGCGTAGTTGG |
| CC77DRAFT_938423  (down) | ADP-ribose pyrophosphatase | CTCCTCGGATGCAAAATGGG | ACGGGTGGTCTGAATTGGAT |
| CC77DRAFT_1063831  (down) | Valine, leucine and isoleucine biosynthesis | CACTGTGAGTAGACGCATGC | ACCAGCATACCTTCTCCACC |
| CC77DRAFT_546152  (down) | Valine, leucine and isoleucine biosynthesis | AGAAAGCAAACCAAGGAGCG | CTTCTCCCGTTCCCAACTCT |
| CC77DRAFT_1061793  (down) | Glycine, serine and threonine metabolism | TGGAAGCTGCTGGATCTCAA | TTCACCCTTTGCGACCTTTG |
| CC77DRAFT_1080602  (down) | Glycine, serine and threonine metabolism | ACGCTCGAAGACATCCAGAA | AACTCAGCGCAGTACTCCTT |
| CC77DRAFT_1019814  (down) | Arginine biosynthesis | GTGTCGCACCCATGATCATC | GCATGCCCTTATGTCCACAG |
| CC77DRAFT_1017719  (down) | Amino sugar and nucleotide sugar metabolism | CGTTTCAATAGGGCACTCGG | GGTGAAGATGAATGCCGTCC |
| CC77DRAFT_1018408  (down) | Alanine, aspartate and glutamate metabolism | CCTTTCTGTCGCATCTACGC | GCGAACGGTACTTGATGACC |
| CC77DRAFT_950966  (down) | Ubiquinone and other terpenoid-quinone biosynthesis | CAACGAGGACTTTGAGGTGC | ATCCTGCTCCCTCTCAATCG |
| CC77DRAFT_1019104  (down) | Ubiquinone and other terpenoid-quinone biosynthesis | CCAATATTCCAGCGCGAACA | GCGGAAATGGGAGGTTGATC |
| CC77DRAFT_1019643  (down) | Endocytosis | GCCTGCCAATACAAGAGACG | CAACCGTGTCATCCAGCAAA |
| CC77DRAFT_1022874  (up) | Autophagy | CAACCTCATCGCTCTTCTGC | AGACATTCGGGAGCCTCAAA |

**Table 2.** Determination of mycelial quantity of *A. alternata* after shaking culture

| Treatment | Mycelial dry weight (g) | Inhibition rate (%) |
| --- | --- | --- |
| *B. tequilensis* + difenoconazole | 0.0908±0.0045 d | 95.33±0.43 a |
| *B. tequilensis* | 0.8122±0.0112 c | 58.29±0.59 b |
| Difenoconazole | 1.3279±0.0098 b | 31.80±0.22 c |
| Blank control | 1.9472±0.0107 a | - |

Note: The mycelial dry weight data are their respective mean ± SD of four replicates.
